# Physiological Levels of *Pik3ca* ^H1047R^ Mutation in the Mouse Mammary Gland Results in Ductal Hyperplasia and Formation of ERα-Positive Tumors

**DOI:** 10.1371/journal.pone.0036924

**Published:** 2012-05-30

**Authors:** Anjali Tikoo, Vincent Roh, Karen G. Montgomery, Ivan Ivetac, Paul Waring, Rebecca Pelzer, Lauren Hare, Mark Shackleton, Patrick Humbert, Wayne A. Phillips

**Affiliations:** 1 Surgical Oncology Research Laboratory, Peter MacCallum Cancer Centre, Melbourne, Victoria, Australia; 2 Sir Peter MacCallum Department of Oncology, University of Melbourne, Parkville, Victoria, Australia; 3 Department of Surgery, St Vincent's Hospital, University of Melbourne, Parkville, Victoria, Australia; 4 Department of Pathology, University of Melbourne, Parkville, Victoria, Australia; 5 Department of Biochemistry and Molecular Biology, University of Melbourne, Parkville, Victoria, Australia; 6 Cell Cycle and Cancer Genetics Laboratory, Peter MacCallum Cancer Centre, Melbourne, Victoria, Australia; 7 Department of Biochemistry and Molecular Biology, Monash University, Clayton, Victoria, Australia; 8 Melanoma Research Laboratory, Peter MacCallum Cancer Centre, Melbourne, Victoria, Australia; Baylor College of Medicine, United States of America

## Abstract

*PIK3CA*, the gene coding for the p110α subunit of phosphoinositide 3-kinase, is frequently mutated in a variety of human tumors including breast cancers. To better understand the role of mutant *PIK3CA* in the initiation and/or progression of breast cancer, we have generated mice with a conditional knock-in of the common activating mutation, *Pik3ca^H1047R^*, into one allele of the endogenous gene in the mammary gland. These mice developed a ductal anaplasia and hyperplasia by 6 weeks of age characterized by multi-layering of the epithelial lining of the mammary ducts and expansion of the luminal progenitor (Lin^−^; CD29^lo^; CD24^+^; CD61^+^) cell population. The *Pik3ca^H1047R^* expressing mice eventually develop mammary tumors with 100% penetrance but with a long latency (>12 months). This is significantly longer than has been reported for transgenic models where expression of the mutant *Pik3ca* is driven by an exogenous promoter. Histological analysis of the tumors formed revealed predominantly ERα-positive fibroadenomas, carcinosarcomas and sarcomas. *In vitro* induction of *Pik3ca^H1047R^* in immortalized mammary epithelial cells also resulted in tumor formation when injected into the mammary fat pad of immunodeficient recipient mice. This novel model, which reproduces the scenario of a heterozygous somatic mutation occurring in the endogenous *PIK3CA* gene, will thus be a valuable tool for investigating the role of *Pik3ca^H1047R^* mutation in mammary tumorigenesis both *in vivo* and *in vitro*.

## Introduction

The phosphoinositide 3-kinase (PI3K)/PTEN/AKT signaling pathway controls a range of fundamental cellular processes which, when de-regulated, are considered hallmarks of cancer. Members of the class 1A PI3K form heterodimers consisting of a regulatory subunit (p85α, p85β or p85γ) and a catalytic subunit (p110α, p110β or p110δ). Among the catalytic subunits, p110α, encoded by the *PIK3CA* gene has been shown to be mutated and activated in many human cancers [1], [2], [3]. Approximately 80% of the mutations identified in this gene are concentrated in three ‘hot spot’ positions, two of which are in exon 9 (E542K and E545K) and one in exon 20 (H1047R).

*PIK3CA* mutations have been observed in up to 40% of breast cancers including both ductal and lobular histological subtypes [1], [4]. *PIK3CA* mutations have also been detected in ductal carcinoma in situ (DCIS), a precursor of breast cancer [5], [6], [7], suggesting they may play a role in tumor initiation rather than progression [7].

The direct impact of *PIK3CA* mutations on clinical outcome remains unclear. While *PIK3CA* mutations have been associated with poor survival [8], [9], [10], others have reported an association with improved outcome [11]. Similarly, while frequently found in estrogen receptor (ER) positive and HER2 positive tumors [12], [13], which tend to have a more favorable prognosis [14], [15], *PIK3CA* mutations also appear to reduce the efficacy of HER2-targeted treatments [16], [17], [18].

*In vitro* studies have demonstrated that the expression of *PIK3CA* mutations in cell lines increase AKT activation and result in morphological changes, increased proliferation and anchorage-independent growth, consistent with an oncogenic role [19], [20], [21]. Moreover, recent mouse models have reported tumor formation following expression of *Pik3ca* mutations in mammary cells [22], [23], [24]. Importantly, these models have used a transgenic approach, where the expression of the mutant protein is under the control of an exogenous promoter potentially leading to its over-expression. Over-expression of wild-type *PIK3CA* has also been shown to be oncogenic [25], [26] and studies on the *K-ras* oncogene have demonstrated that endogenous expression of the *K-ras^G12D^* mutation has very different biological effects compared to transgenic over expression [27].

We have previously generated a novel mouse model with a conditional, Cre recombinase (Cre)-inducible ‘knock-in’ of the *Pik3ca^H1047R^* mutation [28]. Here, we have generated mice with the H1047R mutation knocked into one allele of the endogenous *Pik3ca* gene of the mammary epithelial cell compartment. In this model, expression of the mutant protein is driven by the endogenous promoter and thus is expressed at physiological levels and only in cells that normally express *Pik3ca*. This model accurately reproduces the scenario of a somatic mutation in a single *PIK3CA* allele as occurs in human breast tumors making it ideal for both studying the role of *PIK3CA* mutation in tumourigenesis and for preclinical studies evaluating therapies targeting the PI3K pathway.

## Materials and Methods

### Experimental animals

Mice with targeted expression of a H1047R mutation to one allele of the *Pik3ca* gene of epithelial cells in the mammary gland were generated by crossing a female mouse harboring a latent, Cre-inducible knock-in of the *Pik3ca^H1047R^* mutation (C57BL/6-*Pik3ca^Lat-H1047R^*) [28] with a transgenic male mouse (FVB/N-Tg -MMTV-Cre) expressing Cre under the control of the murine mammary tumor virus (MMTV) promoter [29], [30] obtained from Jane Visvader (WEHI, Melbourne, Australia). Littermates (including wild type, *Pik3ca^Lat-H1047R^* alone, and MMTV-Cre alone) were used as controls. C57BL/6-(ROSA)26Sor mice, which carry a ubiquitously expressed LacZ transgene, in otherwise wild-type mice, were crossed with (FVB/N-Tg-MMTV-Cre) and used for verification of Cre-recombination, by staining for LacZ (β-galactosidase) activity in the harvested mammary gland [31]. Severe combined immunodeficiency (SCID) mice were purchased from the Animal Resource Centre, Canning Vale, Australia.

All experimental procedures involving animals were approved by the Peter MacCallum Cancer Centre Animal Experimental Ethics Committee (AEEC No. E406) and conducted in accordance with the *NHMRC Australian Code of Practice for the Care and Use of Animals for Scientific Purposes*. All mice were maintained and housed in the animal facility of the Peter MacCallum Cancer Centre. All mice were closely monitored and immediately humanely euthanized when the tumors reached a total volume of 1.3 cm^3^ or at any sign of discomfort and/or stress (whichever came first).

### Whole mount/Ductal measurements

Whole inguinal mammary glands from 10 mice of each genotype were dissected out, mounted and spread onto superfrost glass slides, fixed overnight in Carnoy's fixative (ethanol, chloroform, glacial acetic acid 6∶3∶1) followed by rehydration and overnight staining with carmine red stain [1 g carmine red dye (Sigma), 2.5 g potassium alum in 500 ml water]. The mounts were dehydrated, cleared in xylene and mounted in Entellan (HX930910, Merck). The slides were scanned using 4×objectives (IX81, Olympus). The number and size of terminal end buds (TEB) were measured using Meta Imaging series 7.1 (Metamorph, Molecular Devices Corporation). The measured values ± standard deviation was plotted using GraphPad Prism and statistical significance was calculated using Student's t-test.

### Histology and Immunohistochemistry

Formalin-fixed and paraffin-embedded (FFPE) tissue sections were stained with Hematoxylin and eosin (H&E) for histopathological analysis. For immunohistochemistry (IHC) and immunofluorescence (IF) analysis, standard protocols (http:/www.cellsignal.com/support/protocols) were followed. Briefly, antigen retrieval with Tris-EDTA (pH 8) was used for anti-PCNA, keratin 5 (K5), E-cadherin, keratin 8 (K8) and pAKT antibody staining while sodium citrate buffer (pH 6.0) was used for anti-ERα, and pS6 antibodies. Sections (4 µm) were incubated with 3% H_2_O_2_ to inactivate endogenous peroxidase, and blocked with 3% (v/v) pre-immune goat serum (DakoCytomation) in PBS-T (PBS containing 0.05% Tween-20) for 30 min at room temperature. Primary antibodies were applied overnight at 4°C or for 1 hour at room temperature, followed by 1 hour incubation with appropriate secondary antibodies containing different labels as required by IHC and IF procedures. Negative control slides were run with non-immune serum, where possible, or without primary antibody prior to incubation with their corresponding secondary antibody.

For IF, the primary antibodies included K8 (ab104053, Abcam), K5 (PRB16-P, Covance) and E-cadherin (6-10182, BD Biosciences-Pharmingen). While Alexa Fluor® anti-rabbit (A11008, Invitrogen), anti-chicken (A11041, Invitrogen) and anti-mouse antibodies (A31571, Invitrogen) were used as secondary antibodies. Slides were mounted in Prolong® Gold stain with DAPI (P36935, Invitrogen) before imaging using appropriate excitation filters on an Olympus BX-51 fluorescence microscope.

For IHC, the primary antibodies included K8/18 (GP-11, Upstate), p63 (sc-8431, Santa Cruz Biotechnology, Inc.), ER (sc-542, Santa Cruz Biotechnology, Inc.), pAKT S473, (3787, Cell Signaling Technology), pS6 (2211, Cell Signaling Technology), PCNA (6-106-6-5, BD Biosciences). IHC slides were incubated with HRP-labeled anti-mouse or goat anti-rabbit secondary antibodies (DakoCytomation) and visualized using an Envision +Dual link system kit (DakoCytomation). The slides were imaged using ×20 objective and digitally scanned using Aperio digital pathology system (Scan Scope®XT). Nuclear stain scoring such as in the case of anti-PCNA staining was performed using Image Scope software provided with the Aperio scanner.

### β-galactosidase staining

Mammary tissues and mammary whole mounts from (ROSA)26Sor:MMTV-Cre and (ROSA)26Sor control mice were fixed with 2% paraformaldehyde/0.2% glutaraldehyde solution followed by 20% sucrose solution. For histology, tissue was frozen in OCT mounting medium and stored at −80° and 10 µm sections used for staining. Cre-mediated recombination, where present, was identified using X-Gal (V3941, Promega Corp.) and counterstained with nuclear fast red using 10 µm sections [28].

### Mammary epithelial cell preparation

Mammary epithelial cells were isolated from the mammary glands of 6 week old female mice cell using a method previously described [32]. Briefly, after mechanical dissociation of the dissected mammary glands using a scalpel blade, the mammary tissue was placed in culture medium containing 300 U ml^−1^ collagenase (Roche Diagnostics). The resultant organoid suspension was sequentially resuspended in 0.25% trypsin-EDTA for 2 min followed by incubated in buffer containing 5 mg ml^−1^ dispase (Roche Diagnostics) and 0.1 mg ml^−1^ DNase (Worthington) for 5 min. After incubation, the cell pellet was resuspended in NH_4_Cl (0.8%) for 3 min to lyse contaminating red blood cell, then washed with PBS and filtered through a 40-mm mesh. The resulting epithelial cells were either used for labeling and flow cytometry or plated in growth medium (DMEM-F12 containing 1 mM glutamine, 5 µg ml^−1^ insulin, 500 ng ml^−1^ hydrocortisone, 10 ng ml^−1^ epidermal growth factor) supplemented with 5% fetal bovine serum (FBS), for immortalization and generation of cell lines.

### Cell labeling and flow cytometry

Freshly isolated mammary epithelial cells were incubated at 4°C for 30 minutes with appropriate antibodies including PE-CD24 (55326-2, BD Biosciences-Pharmingen), FITC-CD29 (555005BD Biosciences), PECy7-CD31 (56-1410 BD-Pharmingen), PECy7-CD45.1 (56-0578 BD-Pharmingen), bio-CD61 (557853 BD-Pharmingen), PECy7-TER119 (553345 BD-Pharmingen), and APC-Streptavidin (17-4317-82, e-Bioscience. Inc.). After antibody labeling, the cells were washed and resuspended in FACS buffer (PBS+2% FBS) containing viability stain 0.5 µg ml^−1^ propidium iodide, or 10 ng/ml DAPI). The labeled viable single cells were analyzed and sorted using a Fluorescence-activated cell sorter (FACS). Non-epithelial cell populations were excluded from freshly isolated mammary cell suspensions by gating on the cells using well characterized PECy7-conjugated endothelial (CD31) and hematopoietic (CD45 and TER119) antigens. As previously detailed [32], the remaining live CD45^−^CD31^−^TER119^−^ (Lin^−^) cells were sorted based on FITC-CD29, PE-CD24 and APC-CD61 labeled cells and collected using FACSDiVa or FACSAria™II (Becton Dickinson). Consequently, the Lin^−^CD29^hi^CD24^+^ cells represent the basal cell population (enriched for stem cells, which possess multi-lineage differentiation capacity) while Lin^−^CD29^lo^CD24^+^ cells represent the luminal population. The luminal population was further sorted on the basis of CD61 expression where Lin^−^CD29^lo^CD24^+^CD61^+^ cells represent the luminal progenitor cell population [33]. After sorting the cells were routinely re-analyzed to confirm purity and viability were greater than 95%.

### 2D proliferation assay for FACS sorted cells

For colony assays, FACS sorted cells were plated into duplicate wells of 12-well plates containing a feeder layer of irradiated NIH-3T3 cells. Cells were allowed to form colonies in growth medium containing 20 ng ml^−1^ of cholera toxin for 7 days before staining with 0.05% crystal violet. Numbers of colonies were counted and plotted using GraphPad Prism.

### Generation of immortalized mammary epithelial cell lines

Mammary epithelial cells from 6 week old mice were isolated and immortalized using dominant-negative p53 and E1A as previously described [34]. In the first instance, we isolated and immortalized mammary epithelial cells from mice heterozygous for the latent mutant allele (*Pik3ca^Lat-H1047R^*) and induced the mutation by infection with a retrovirus expressing a pMSCV-Cre-IRES-GFP construct (a kind gift of Kay Macleod, University of Chicago, USA) [35] while MSCV-IRES-GFP infected cells were used as controls. Transduced cells were enriched by using FACS to select for GFP positive cells.

We also prepared mammary epithelial cells from mice already expressing the *Pik3ca^H1047R^* mutation (*Pik3ca^H1047R^*: MMTV-Cre mice) and wild type controls (MMTV-Cre only mice). These cells were either used immediately as primary cells or immortalized with dominant-negative p53/E1A as described above.

All cell lines were maintained in (DMEM-F12 containing 1 mM glutamine, 5 µg ml^−1^ insulin, 500 ng ml^−1^ hydrocortisone, 10 ng ml^−1^ epidermal growth factor) supplemented with 5% FBS, and cultured at 37°C in 5% CO_2_. Knock-in of the mutation was confirmed in all cell lines by cDNA sequencing.

### *In vitro* experiments using iMMECS

The proliferative potential of mutant cells compared to control cells was assessed by direct cell count using a coulter counter or by confluence measurements using an IncuCyte™ live cell imaging system. For analysis of tumor forming potential, 3×10^6^ freshly isolated primary mammary epithelial primary cells, or immortalized cell lines, in 30 µl of a mixture of PBS and 50% Matrigel (1∶1), were injected into the inguinal mammary fat pad of 4–6 week old SCID mice. Tumor growth was monitored using calipers and the mice sacrificed and tumors harvested when the tumor burden reached >1.4 cm^3^ or at signs of severe illness (hunching, ruffled fur or distress).

### Statistics

All statistical analyses were performed using GraphPad Prism 5 software. Statistical analyses were performed using a 2-tailed Student's t-test or Log-rank (Mantel-Cox) Kaplan-Meier survival test, as appropriate. *p* values<0.05 were considered statistically significant.

## Results

### Expression of *Pik3ca^H1047R^* in the mouse mammary gland

**Figure 1:**
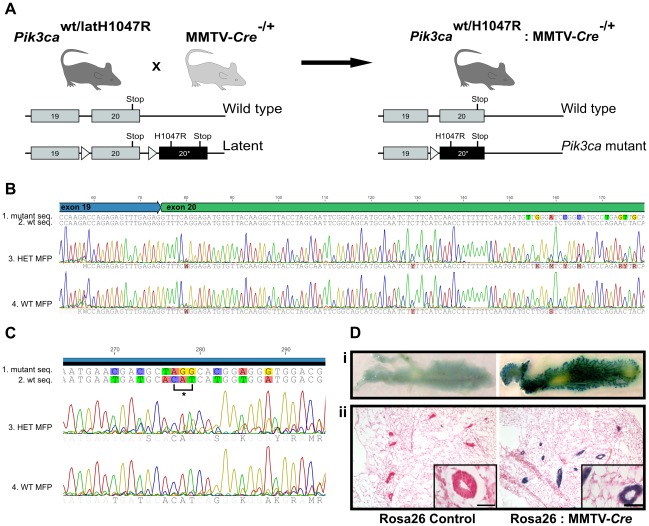
*Pik3ca* ^H1047R^ inducible knock-in mouse model. (A) A schema outlining the generation of mice expressing *Pik3ca* ^H1047R^ in the mammary gland. Mice heterozygous for the latent mutant allele (*Pik3ca* ^wt/LatH1047R^) were crossed with mice heterozygous for the transgenic MMTV–Cre allele (MMTV-Cre^−/+^). Cre-mediated recombination results in the excision of everything between the LoxP sites (triangles) allowing the mutant version of exon 20 (dark rectangle) to replace the wild type exon 20, thus generating mice that express the *Pik3ca* ^H1047R^ mutation in mammary epithelial cells. (B) Cre-mediated knock-in of the mutant exon 20 was confirmed by sequencing RNA extracted from the mammary fat pad (MFP) from heterozygous *Pik3ca* ^H1047R^:MMTV-Cre (HET) and *Pik3ca^wt^* (WT) mice. RNA extracted from WT and HET MFP was reverse transcribed into cDNA and sequenced from exon 19 into exon 20 (exon 19-specific primer: 5′-CAAGAGTACACCAAGACCAGAGAGTT-3′) and confirmed by sequencing in the reverse direction from exon 20 (5′- TCCAATCCATTTTTGTCGTCC-3′). Shown are the expected sequences (1, 2) and the actual sequence traces (3, 4) for cDNA from WT MFP (*Pik3ca* control) (1, 3) and HET-MFP (*Pik3ca* mutant) (2, 4). Note that the representative cDNA sequence from the HET-MFP is heterozygous at the sites of silent base changes engineered into the mutant exon 20 [28] confirming successful knock-in of the *Pik3ca* ^H1047R^ mutation in HET MFP. (C) The section of the sequencing trace from (B) showing the region spanning position 1047 showing the bases changes responsible for the His→Arg substitution (*). (D) Whole mounts (i) and 10 µm sections (ii) of mammary tissue from (ROSA)26Sor control mice (left) and (ROSA)26Sor:MMTV-Cre mice (right) were stained using X-Gal and counterstained with nuclear fast red. MMTV-Cre-mediated recombination, where present, is identified by blue staining. Scale bar, 50 µm.

To examine the role of *Pik3ca^H1047R^* mutation during mammary development and its ability to induce mammary tumors, we targeted the mutation to the mammary gland. Mice harboring a latent Cre-inducible *Pik3ca^Lat-H1047R^* allele [28] were crossed with mice expressing Cre under the control of the MMTV promoter [29] resulting in mice heterozygous for *Pik3ca^H1047R^* expression in the mammary gland (Figure 1A). Successful knock-in and mutation expression was confirmed by sequencing of RNA isolated from the mammary gland of mutant mice (Figure 1B, C). No mutation was detected in RNA isolated from wild-type mice (Figure 1B, C). Similarly no mutation was detected in MMTV-Cre alone or *Pik3ca^Lat-H1047R^* alone control mice (data not shown). Mammary epithelial cell expression of Cre driven by MMTV was confirmed by crossing MMTV-Cre mice with a transgenic mouse bearing a Cre-inducible *LacZ* allele at the (ROSA)26Sor locus (Figure 1D).

### *Pik3ca^H1047R^* expression in the developing mammary gland results in enhanced ductal morphogenesis

**Figure 2:**
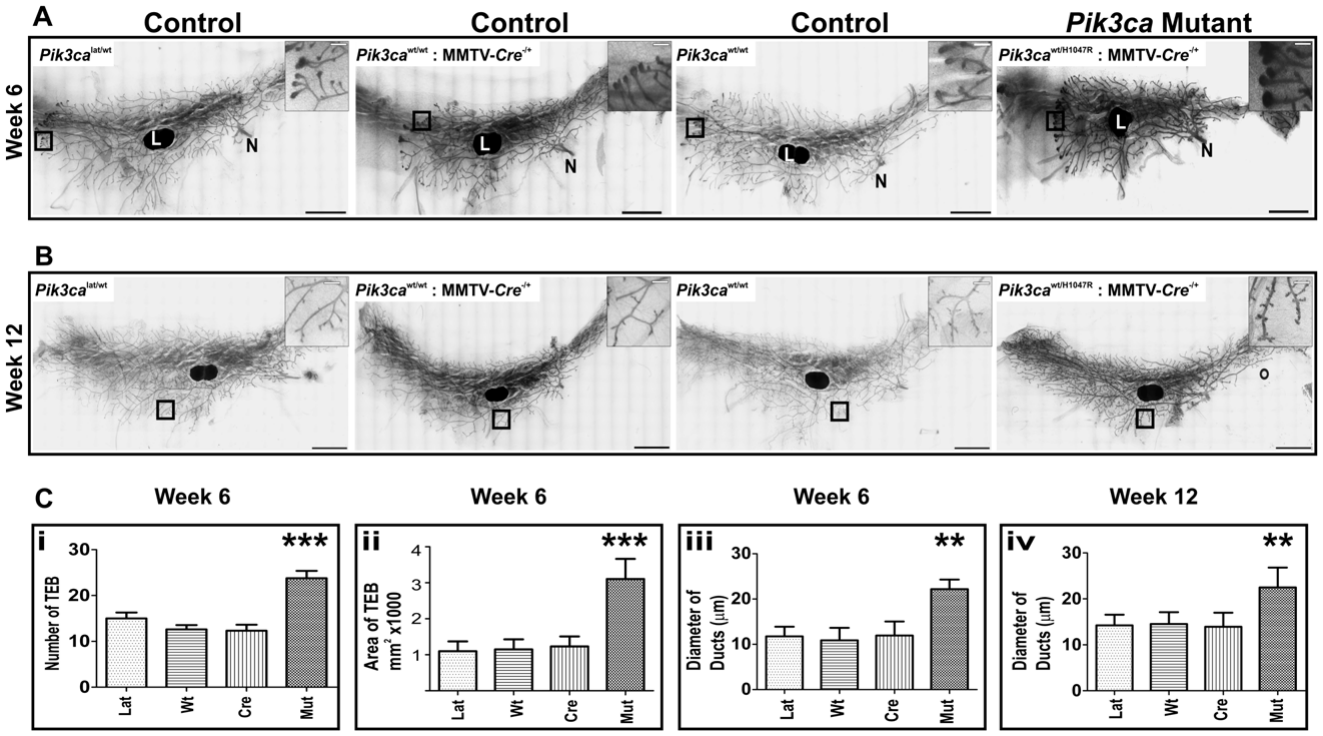
Expression of *Pik3ca* ^H1047R^ in developing mammary gland increases ductal morphogenesis. Mammary gland whole mounts from mice at 6 weeks (A) or 12 weeks (B) of age were stained with carmine red and visualized by scanning microscopy. Shown are representative whole mounts from mutant expressing *Pik3ca* ^H1047R^:MMTV-Cre (*Pik3ca* mutant) mice as well as from wild type, *Pik3ca* ^Lat/H1047R^ alone and MMTV-Cre alone, control mice. N, nipple. L, lymph node. Scale bar, 500 µm. Insets show higher magnification of boxed region (scale bar of insets, 50 µm). (C) Morphometric analysis using MetaMorph software was used to quantify (i) the number of terminal end buds (TEB) in the mammary gland of 6 week old mice (n = 10), (ii) the area of the ten largest TEB in 6 week old mice (n = 10 mice). The diameter of 20 randomly selected ducts (n = 5 mice), was measured at a point equi-distant from the nipple, in control and mutant-expressing mammary glands from 6 week old (iii) and 12 week old (iv) mice. Shown are mean ± SD. Lat, *Pik3ca* ^LatH1047R^ alone; Wt, wild type; Cre, MMTV-Cre alone; and Mut, *Pik3ca* ^H1047R^: MMTV-Cre. ** denotes p<0.005; *** denotes p<0.0001 (two tailed t-test) between *Pik3ca* mutant and any of the control group.

We examined the effect of *Pik3ca^H1047R^* expression on the mammary gland at various developmental stages including puberty (6 weeks of age) and early adulthood (12 weeks) and during pregnancy (day 14) and involution (5 days post weaning). Mutant female mice could produce milk and suckle the pups as normal. Also, the pups of the *Pik3ca^H1047R^* mutant mothers were healthy and gained weight similar to the pups from the control mothers, suggesting that expression of the *Pik3ca^H1047R^* mutation did not adversely effect lactation. At the macroscopic level, *Pik3ca^H1047R^* expressing mammary glands appeared to develop normally. However, microscopic examination of mammary gland whole mounts showed that *Pik3ca^H1047R^* expression altered ductal morphogenesis, as evidenced by increased number and size of invading terminal end buds (TEB) at 6 weeks (Figure 2A, Ci and Cii), and increased secondary and tertiary branching at 12 weeks of age (Figure 2B). Furthermore, the ducts of the *Pik3ca^H1047R^* expressing mammary glands at both 6 and 12 weeks were noticeably dilated (Figure 2A, 2B) with significantly larger cross sectional diameter (Figure 2Ciii, Civ respectively). Interestingly, during pregnancy (day14) or involution (day5) the mammary glands of *Pik3ca^H1047R^* mutant mice showed no noticeable difference compared to control mice (data not shown).

**Figure 3:**
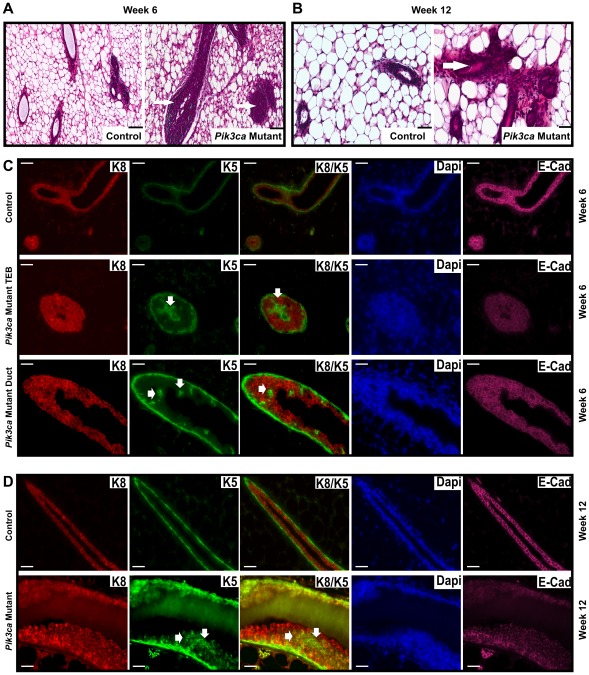
Expression of *Pik3ca* ^H1047R^ in developing mammary gland induces ductal hyperplasia. (A, B) Formalin-fixed paraffin-embedded sections of mammary glands from control and mutant (*Pik3ca* ^H1047R^–expressing) mice were stained with hematoxylin and eosin (H&E). Shown are representative sections showing the presence of a multilayered epithelium (arrow) in the ducts of *Pik3ca* ^H1047R^ –expressing mammary gland from 6 week old mice (A) and regions of hyperplastic epithelium (arrow) in the *Pik3ca* ^H1047R^ expressing mammary gland of 12 week old mice (B). Sections from control and mutant mammary glands are pictured at same magnification (scale bar 50 µm). (C, D) Representative sections were also stained for K5 (green), K8 (red) and E-cadherin (pink) using immunofluorescence. DAPI (blue) was used to stain nuclei. Note that, in addition to staining the basal layer of the epithelium, K5 also stains anaplastic cells (arrows) in the terminal end buds (TEB) and ducts of the *Pik3ca* ^H1047R^ –expressing mammary gland of 6 week (C) or 12 week mice (D). All sections were imaged at the same magnification using appropriate excitation filters. Scale bar, 50 µm.

Histological examination of mammary glands from 6 week old mice showed that, while most ducts in the mammary glands of control mice had a clear lumen, the lumen of the ducts in *Pik3ca^H1047R^* mutant mammary glands were filled with cells (Figure 3A). Similarly, the majority of ducts in 12 week old mutant mice were enlarged compared to wild type controls and displayed areas of multilayered epithelium, which was hyperplastic in some regions, and showed presence of an eosinophilic luminal secretion (Figure 3B). In addition, the mutant ducts were surrounded by an increase in number of stromal cells, as evident by eosin staining, a phenotype that was not observed in the ducts of control mice. The increased periductal stroma stained positively for α-smooth muscle actin (Supplementary Figure S1A) but showed no reactivity to the epithelial cell markers p63, K5 or K8 (data not shown). Interestingly, enhanced PI3K pathway activity, as assessed by immunohistochemistry for pAKT or pS6, was not evident at these early developmental time points (6 and 12 weeks).

Co-immunofluorescence using anti-K5, anti-K8 and anti-E-cadherin antibodies, together with DAPI to stain the nuclei, was used to study the ductal epithelium of the *Pik3ca^H1047R^* mice. In the normal mammary gland from 6 week old mice K5 stained the outer epithelial layer of the duct and TEB cap cells. However, in the *Pik3ca^H1047R^* mutant mammary gland, in addition to staining the outer epithelial cell layer of TEB and ducts, K5 positive cells were also present in the body of the TEB and in luminal epithelial layers of the ducts (Figure 3C, thick arrows), suggesting anaplasia of basal cells in the mutant mammary gland. Anaplasia of basal cells was confirmed by anti-p63 immunohistochemistry (arrow, Supplementary Figure S1B). As expected, K8 positive cells lined the lumen of both the control and the mutant mice (Figure 3C). In particular, the multilayered epithelium of the *Pik3ca^H1047R^* mutant ducts was K8 positive (Figure 3C). Immunohistochemistry confirmed the majority of multilayered epithelium stained positively for anti-K8/18 antibody (Supplementary Figure S1C). E-cadherin immunostaining showed that K8 positive cells mostly co-stained for E-Cadherin. While E-cadherin was present in the lateral walls in most of the cells of the mammary ducts from mutant mice, although some ducts also showed diffused and/or reduced lateral cell wall staining, suggestive of some loss of polarity as early as six weeks (Figure 3C). Loss of polarity in the epithelium was confirmed at 6 weeks by anti-β-Catenin immunostaining showing reduced membrane staining and increased nuclear staining in many of the ducts from *Pik3ca^H1047R^* mutant mice (Supplementary Figure S1D). Similar results were observed in mammary glands from 12 week old mice (Figure 3D).

### *Pik3ca^H1047R^* expression results in expansion of the luminal progenitor population

**Figure 4:**
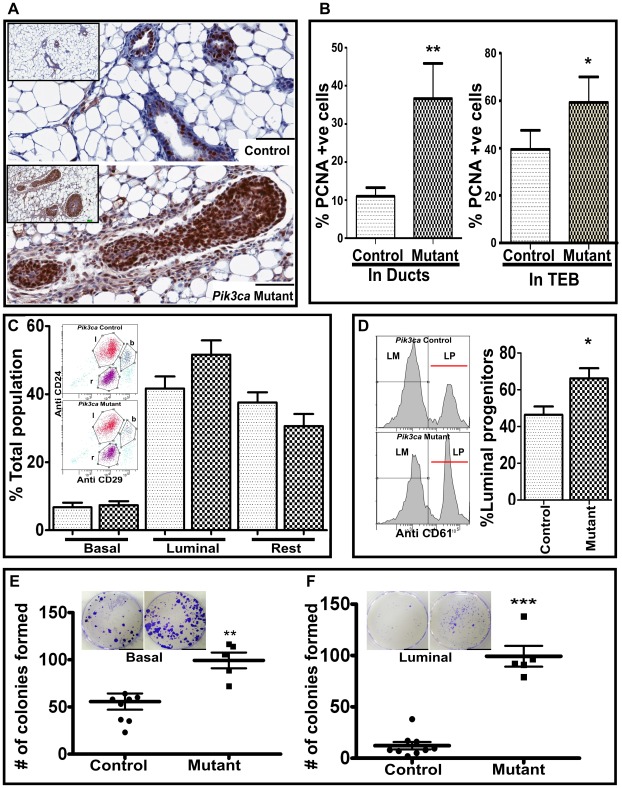
*Pik3ca* ^H1047R^ increases cell proliferation and enrichment of luminal progenitors *in vivo*. (A) Representative sections of control and mutant (*Pik3ca* ^H1047R^-expressing) mammary glands from 6 week old mice stained with anti-PCNA antibody. Scale bar, 100 µm. Insets show lower magnification image of surrounding region. (B) The percentage of PCNA positive cells in ducts and terminal end buds (TEB) of control and mutant mammary glands. Shown are mean ± SEM from 3 mice (7 TEB and 7 ducts per mouse). * indicates p<0.05 and ** indicates p<0.005 compared to respective controls (two tailed t-test). (C) Freshly isolated Lin^−ve^ epithelial cells from the mammary glands of 6 week old mice were sorted on the basis of CD24 and CD29 expression (insets) and the percentage of cells in the Basal (b, Lin^−ve^CD29^hi^CD24^+^), Luminal (l, Lin^−ve^CD29^lo^CD24^+^) and the ‘Rest’ (r) populations calculated. Shown are mean ± SEM for *Pik3ca* ^H1047R^ mutant (n = 5) and *Pik3ca* ^Wt^ control (n = 9). (D) The luminal cell population was further sorted on basis of CD61 to generate a luminal progenitor (LP, Lin^−ve^CD29^lo^CD24^+^ CD61^+^) and non-progenitor (LM) populations. Shown are mean ± SEM for *Pik3ca* ^H1047R^ mutant (n = 5) and *Pik3ca* ^Wt^ control (n = 9). (E&F) The various cell populations (1×10^3^ cells) were plated on a layer of irradiated NIH3T3 cells and after 9 days stained with 0.05% crystal violet. The number colonies formed by Lin^−ve^CD29^hi^CD24^+^(Basal) (E) and Lin^−ve^CD29^lo^CD24^+^ CD61^+^ (luminal progenitor) (F) populations were counted. Shown are mean ± SEM for Pik3ca^H1047R^ mutant (n = 5) and Pik3ca^Wt^ control (n = 9). ** indicates p<0.005 and *** indicates p<0.001 compared to respective controls (two tailed t-test).

The increased cell number observed within the epithelium of the ducts and TEB expressing *Pik3ca^H1047R^* did not appear to be due to reduced apoptosis as there was no significant difference in TUNEL staining when compared to control mammary glands (data not shown). However, increased proliferation within the multilayered epithelia of the *Pik3ca^H1047R^* expressing ducts and TEB was detected by anti-PCNA staining (Figures 4A, 4B).

In order to explore the origin of cells in the multilayered epithelium, we used FACS analysis to isolate the various subpopulations of the mammary epithelial cells. Epithelial cells from control and *Pik3ca^H1047R^* expressing mammary glands from 6 week old mice were isolated and sorted using anti-CD29 and anti-CD24. While the FACS profiles for the *Pik3ca^H1047R^* expressing and wild type cells were similar (insert on Figure 4C), the Lin^−^CD29^lo^CD24^+^ (luminal cell) population was increased in the *Pik3ca^H1047R^* expressing mice as compared to control mice (Figure 4C). Furthermore anti-CD61 antibody was used to separate the luminal cells into mature and progenitor populations. A significant increase in the number of Lin^−^CD29^lo^CD24^+^CD61^+^ (luminal progenitors) was observed in the *Pik3ca^H1047R^* expressing mice (Figure 4D). We also found that when allowed to form colonies on an irradiated feeder layer of cells, colony forming ability of either Lin^−^CD29^hi^CD24^+^ (the stem cell enriched basal population) or Lin^−^CD29^lo^CD24^+^CD61^+^ (luminal progenitors) was significantly enhanced in the cells from *Pik3ca^H1047R^* mutant mice as compared to cells from control mice (Figure 4E, F). In addition the size of the colonies formed by either cell population from mutant mice was larger than the colonies from wild type mice (inserts in Figure 4E, F). Neither the mature luminal cells nor the remaining (rest) cell populations from either the mutant or control mammary glands formed any colonies in similar assay.

### *Pik3ca^H1047R^* expression leads to mammary gland hyperplasia and tumorigenesis

**Figure 5:**
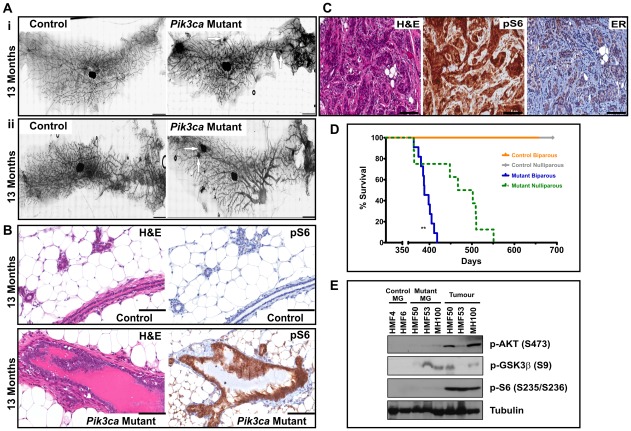
Expression of *Pik3ca* ^H1047R^ induces pre-neoplastic lesions and tumorigenesis in aged mice. (A). Representative whole mounts of control (MMTV-Cre alone) and mutant (*Pik3ca* ^H1047R^:MMTV-Cre) mammary glands from 13 month old nulliparous (i) and biparous (ii) mice. Arrows indicate foci of sclerosis adenosis. Scale bars, 500 µm. (B) Representative sections from control (MMTV-Cre alone) and mutant (*Pik3ca* ^H1047R^:MMTV-Cre) mammary glands from 13 month old biparous mice were stained with H&E or anti-pS6 antibody. Scale bars, 100 µm. (C) Representative sclerosing adenosis lesion from *Pik3ca* ^H1047R^-expressing mammary glands from aged (>12-month) old mice were stained with H&E, and with anti-pS6 and anti-ERα antibodies. Scale bars, 100 µm. (D) Kaplan-Meier survival curves for aged control and mutant (*Pik3ca* ^H1047R^:MMTV-Cre) mice. Shown are nulliparous (green broken line, n = 10) and biparous (blue solid line, n = 11) and respective control mice (nulliparous, gray line, n = 30; biparous, orange line, n = 10). ** The median tumor free survival in biparous mice was significantly (p<0.005) reduced compared to nulliparous mice. (E) Protein lysates from control and mutant mammary glands and tumor tissue from nulliparous aged (>12 month) mice were separated by SDS-PAGE and immunoblotted for analysis of PI3K pathway activation using anti-pAKT, anti-pGSK3β, and anti-pS6, as well as anti-tubulin (loading control). Immunoreactive proteins were detected with horse-radish peroxidase conjugated secondary antibodies and visualized with enhanced chemiluminescence. Samples are identified by their corresponding mouse ID. MG, mammary gland.

Cohorts of *Pik3ca^H1047R^* expressing and wild type female (nulliparous and biparous) mice were set aside for ageing and followed for tumor development. Examination of mammary whole mounts from 13 month old nulliparous (Figure 5Ai) and biparous (Figure 5Aii) mice revealed enlarged ducts, particularly in the nipple region of the biparous mice (Figure 5Aii), and small focal lesions in the mutant glands (arrowed in Figures 5Ai; Aii). Histological examination revealed the mammary glands of aged mutant mice had ectatic ducts filled with eosinophilic luminal secretions (Figure 5B). However, unlike at earlier time points, the activation of PI3K pathway in the ductal cells of aged mutant mice was detectable using pAKT (data not shown) or pS6 staining (Figure 5B). Two microscopic foci of sclerosis adenosis and one small intraductal fibroadenoma were noted in the *Pik3ca^H1047R^* expressing glands (Figure 5C). Activation of the PI3K pathway was evident in the area of sclerosis adenosis as indicated by increased expression of pAKT (not shown) and also pS6 (Figure 5C). These foci were also ERα positive (Figure 5C).

Ageing mice beyond 12 month resulted in tumor formation in the mutant mice cohort. As shown in Figure 5D, all nulliparous and biparous *Pik3ca^H1047R^* expressing female mice were culled due to the development of mammary tumors. While the nulliparous cohort had a median tumor-free survival of 484 days, the tumor formation was significantly accelerated in biparous mutant mice with a median tumor-free survival of 393 days. All mice were otherwise disease free and all other tissues showed normal histology in both mutant and control mice. No macroscopic metastasis was found under these experimental conditions; however it should be noted that the mice were culled when the primary tumors reached a predetermined, ethically-limiting, size. Sequencing analysis of RNA confirmed the presence of the *Pik3ca^H1047R^* mutation in all tumors.

**Figure 6:**
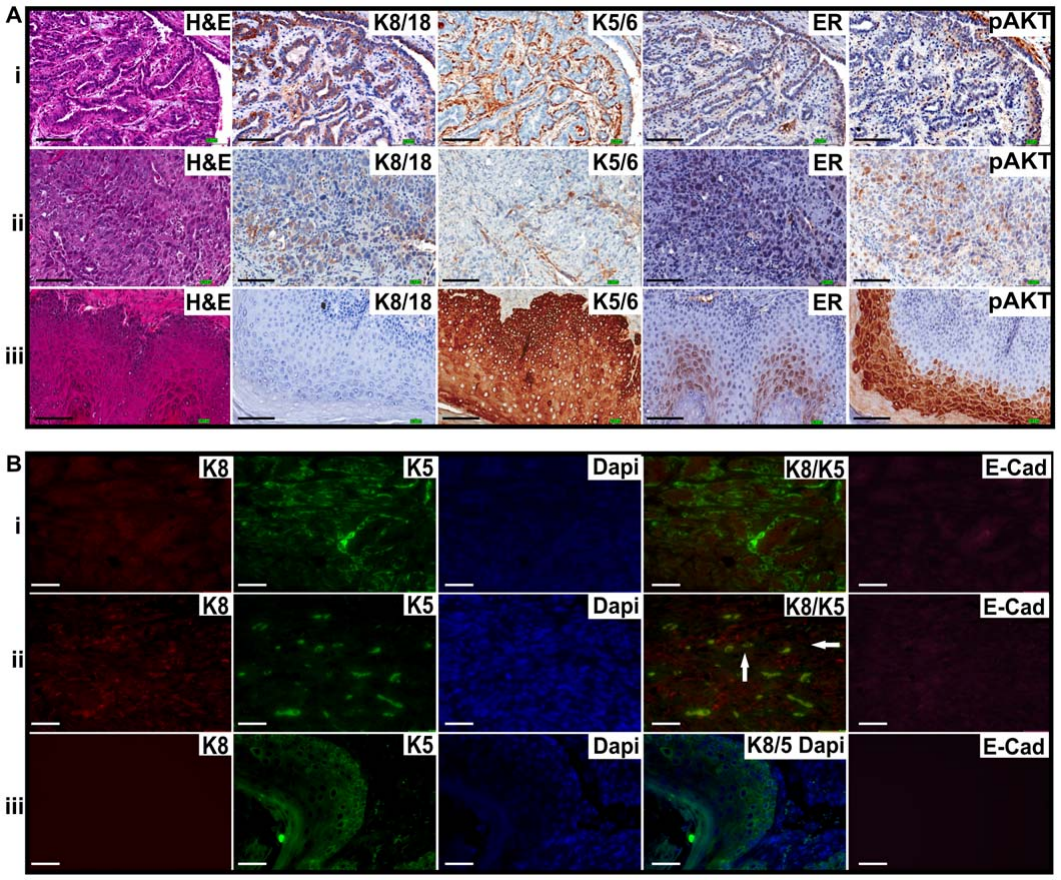
*Pik3ca* ^H1047R^ tumors express basal-like and luminal-like markers. (A) Mammary tumors from *Pik3ca* mutant mice were stained with H&E or immunohistochemically stained with anti-K8/18, anti-K5/6, anti-ERα or anti-pAKT(Ser473). Shown are representative examples of a benign fibroadenoma (i), carcinosarcoma (ii), and the squamous elements of an adenosquamous carcinoma (iii). Scale bar is 100 µm. (B) Mammary tumors from *Pik3ca* mutant mice were stained for immunofluorescence using anti-K8 (red), anti-K5 (green), or anti-E-cadherin (pink). Dapi (blue) was used to stain nuclei. Shown are representative examples of fibroadenoma (i), carcinosarcoma (ii), and the squamous elements of an adenosquamous carcinoma (iii). The presence of a few K5 and K8 double-positive cells can be seen in the carcinosarcoma (arrows, middle panel). Scale bar is 50 µm.

Histopathological assessment of the tumors revealed that they were a mix of benign fibroadenomas (45%), carcinosarcomas or sarcomas (42.5%), adenoquamous carcinomas (10%) and an osteosarcoma (2.5%) (Figure 6Ai–iii). Due to the frequent occurrence of biphasic tumors with both epithelial and mesenchymal elements (fibroadenomas and carcinosarcomas), we investigate the potential role of bipotent progenitor cells in tumor formation and the effect of *Pik3ca* mutation on epithelial–mesenchymal transition (EMT). Immunofluorescence and immunohistochemical analysis of tumor sections revealed the presence of basal-like (K5/6 positive) and luminal-like (K8/18 positive) epithelial cells in fibroadenomas (Figure 6Ai) and carcinosarcomas (Figure 6Aii) while the squamous elements of the adenosquamous carcinomas (Figure 6Aiii) contained only K5/6 positive cells. Notably, some fibroadenomas and carcinosarcomas contained cells that showed co-localization of K5 and K8 staining. Furthermore, while some of the ductal structures and K8 positive cells retained E-cadherin expression in lateral junctions, presumably due to normal ducts trapped in the tumor mass, most K8 positive cells showed diffuse or reduced E-cadherin staining. On the other hand, the adenosquamous carcinomas stained positively for K5 but did not express K8 and E-cadherin (Figure 6B, iii).

Activation of PI3K pathway in tumors was confirmed by immunostaining for pAKT (Figure 6A) and pS6 (data not shown) and western blotting for pAKT and pS6 (Figure 5E). GSK3β was also phosphorylated in these tumors, as well as in non-tumor mammary gland tissue from the *Pik3ca^H1047R^* mutant mice (Figure 5E).

### Cells from *Pik3ca^H1047R^*-induced tumors maintain malignant potential

**Figure 7:**
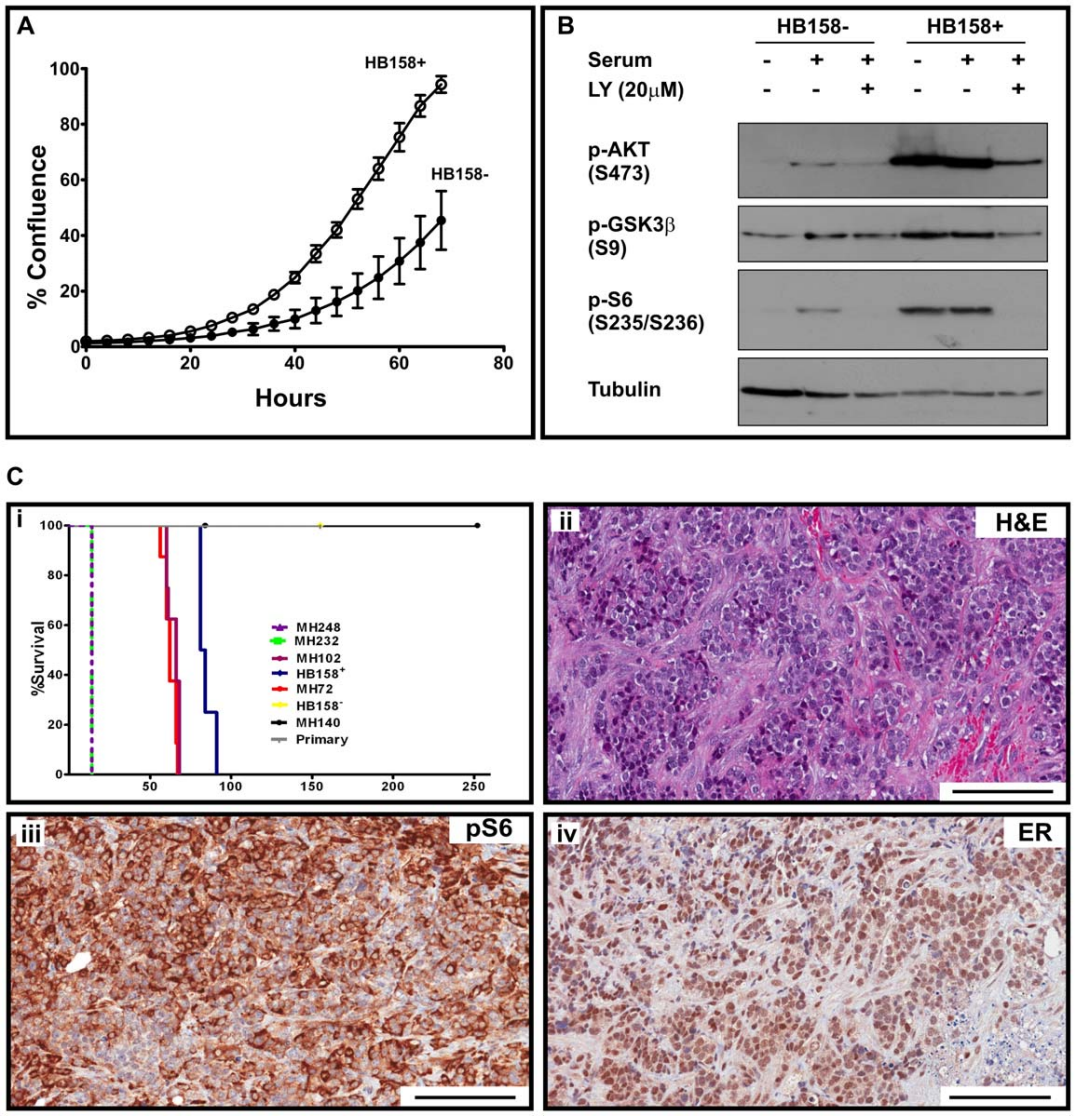
Effects of *Pik3ca* ^H1047R^ expression on *in vitro* proliferation and tumorigenicity of mammary epithelial cells. Isogenic mammary epithelial cells expressing wild type or mutant *Pik3ca* were generated by isolating mammary epithelial cells from a *Pik3ca^Lat-^* ^H1047R^ mouse and immortalizing them with dominant-negative p53 and E1A. Expression of the mutant protein was induced using pMSCV-Cre-IRES-GFP and GFP positive cells were isolated by FACS. Control cells expressing wild type *Pik3ca* were generated by infection with pMSCV-IRES-GFP retrovirus. (A) Wild type (HB158^−^) or mutant (HB158^+^) mammary epithelial cells were seeded into 12-well plates at low density (20,000 cells) and cell growth monitored over 68 hours using an IncuCyte™ live cell imager to assess cell confluence. Shown are mean ±SD from a representative experiment performed in triplicate. (B) Isogenic wild type (HB158^−^) or mutant (HB158^+^) cells were starved for 3 hours and then stimulated with 20% FBS for 15 minutes. Where indicated, cells were treated with 20 µM of the PI3K inhibitor LY-294002 (LY) added at time of starvation. Protein lysates were separated by SDS-PAGE and immunoblotted with anti-pAKT, anti-pGSK3β, anti-pS6, and anti-Tubulin (loading control). (C) Immortalized wild type (HB158^−^) and mutant (HB158^+^) mammary epithelial cells (3×10^6^ cells) were injected into mammary fat pads of immunocompromised (SCID) recipient mice. Shown is the Kaplan-Meier survival curve for injected mice (i) and representative sections from the undifferentiated carcinomas, formed by the *Pik3ca* ^H1047R^-expressing cells (HB158^+^), stained with H&E (ii), anti-pS6 (iii) or anti-ERα (iv). Scale bars, 100 µm.

To examine whether cells from *Pik3ca^H1047R^*-induced tumors could maintain their malignant potential, pieces of the *Pik3ca^H1047R^* tumors were disaggregated and allowed to grow in 2D culture, resulting in the generation of cell lines without need for immortalization. When injected into the mammary fat pad of SCID mice these cell lines (MH232 and MH248) formed tumors within 14 days (Table 1 and Figure 7Ci).

**Table 1:** Immortalized mammary epithelial and mammary tumor cells expressing *Pik3ca* ^H1047R^ mutation form tumors when injected into the mammary fat pad of SCID mice.

Cells injected	No. of mice injected	No. of mice with tumor development
MH 232 (*Pik3ca^H1047R^*; mouse tumor)	12	12
MH 248 (*Pik3ca^H1047R^*; mouse tumor)	12	12
HB158^−^ (*Pik3ca^Lat H1047R^*; control)	8	None
HB 158^+^ (*Pik3ca^H1047R^*; mutant)	8	8
Primary (MMTV-Cre; control)	6	None
Primary (*Pik3ca^H1047R^*: MMTV-Cre; mutant)	6	None
MH 140 (MMTV-Cre; control)	8	None
MH 72 (*Pik3ca^H1047R^*; mutant)	8	8
MH 102 (*Pik3ca^H1047R^*; mutant)	8	8

### *In vitro* knock-in of the *Pik3ca^H1047R^* mutation induces transformation of immortalized mammary epithelial cells

To test the tumorigenic potential of the *Pik3ca^H1047R^* mutation in vitro, we prepared immortalized mammary epithelial cells from mice heterozygous for the latent mutant allele (*Pik3ca^Lat-H1047R^*) and induced the knock-in of the mutation by infection with a Cre-expressing retrovirus (pMSCV-Cre-IRES-GFP). The resulting *Pik3ca^H1047R^* expressing cells (HB158^+^) grew faster than cells infected with the control retrovirus pMSCV-IRES-GFP (HB158^−^) (Figure 7A) and exhibited constitutive activation of the PI3K pathway as evidenced by increased pAKT, pGSK3β and pS6 levels, both with and without serum stimulation (Figure 7B). The *Pik3ca^H1047R^* expressing cells (but not the un-induced controls) were also tumorigenic forming tumors when injected into the mammary fat pads of immunocompromised mice (Table 1 and Figure 7Ci). Analysis of tumor sections showed undifferentiated carcinomas that had an activated PI3K pathway, as shown by pS6 positivity (7Ciii), and were ERα-positive (Figure 7Civ).

We also examined the tumor-forming capacity of immortalized *Pik3ca^H1047R^* expressing mammary cells prepared from *Pik3ca^H1047R^*: MMTV Cre mice. Similar to HB158^+^, the *Pik3ca^H1047R^*-expressing cell lines (MH72 and MH102) grew quicker than the non-expressing control cells (MH140) in 2D culture (data not shown) and formed tumors when injected into immunocompromised mice (Table 1). No tumors were formed from the control cells. Interestingly, primary (non-immortalised) *Pik3ca^H1047R^* expressing cells failed to produce tumors in immunocompromised mice (Table 1 and Figure 7Ci) suggesting that *Pik3ca^H1047R^* expression alone is insufficient to induce transformation of mammary epithelial cells *in vitro*.

## Discussion

Mutations in the *PIK3CA* gene are found in up to 40% of human breast cancers [1], [4] and *in vitro* expression of these mutations in cell lines has suggested that they are oncogenic [20], [36]. Here we have generated mice with a conditional knock-in of the *Pik3ca^H1047R^* mutation targeted to the mammary gland, in order to investigate the role of this mutation in mammary tumorigenesis *in vivo*.

In contrast to other recently described models that take a transgenic approach, where expression of the mutant allele driven by an exogenous promoter [22], [23], [24] we have inserted the mutation into the endogenous *Pik3ca* gene so that expression of the mutant allele is under the control of the endogenous promoter. As a result, the mutation is expressed at physiological levels and only in cells that would normally express *Pik3ca* thus mimicking the scenario of a heterozygous somatic mutation in the endogenous gene, as occurs in human tumors.

We found that expression of *Pik3ca^H1047R^* in the epithelial cells of the mammary gland results in an epithelial hyperplasia detectable by 6 weeks of age. Such early changes suggest that the *Pik3ca* mutation may be an early event in breast cancer development, a result consistent with findings of *PIK3CA* mutations in DCIS [5], [6], [7]. Increased proliferative capability of mammary epithelial cells, as a consequence of *Pik3ca* mutation, potentially makes the cells susceptible to further mutations. Increasing evidence shows that early genetic alterations in mouse mammary gland progenitor or luminal cells, which result in changes such as hyper-proliferation, are involved in tumor initiation [37], [38], [39], [40], [41].

We found increased ductal morphogenesis in the *Pik3ca^H1047R^*-expressing mammary glands of pubertal and young adult mice. This is consistent with results showing that activation of the PI3K/AKT pathway, by activation of receptor tyrosine kinases or loss of PTEN, has been linked to increased mammary duct growth and branching [42], [43], [44], [45]. Also, inhibition of PI3K activity has been shown to block both ductal elongation and branching [46], [47] suggesting a role for the PI3K/AKT pathway in this process. However, expression of a constitutively activated Akt in the mouse mammary gland shows no major changes in ductal morphogenesis [48], [49], suggesting the possibility that the ductal morphogenesis observed in the developing *Pik3ca* mutant mammary glands might be controlled by AKT independent pathways [49].

*Pik3ca^H1047R^* expressing mice eventually formed mammary tumors but only after a long latency of 12–18 months. The development of tumors was significantly accelerated by pregnancy but the mean survival for biparous mice was still more than 12 months. In human cancer, *PIK3CA* mutations are detected in DCIS [5], [6], [7] suggesting that *PIK3CA* mutation is an early event in the tumorigenic process and that, presumably, other mutational events are required to initiate progression to invasive carcinoma. Similarly, hyperplastic changes induced by *Pik3ca^H1047R^* expression alone, as early as 6 weeks of age, are consistent with the earliest events in the tumorigenic process. The long latency for tumor formation may therefore suggest a secondary event is required for progression to cancer.

Interestingly, we could not detect enhanced activity of the PI3K/AKT pathway in the *Pik3ca^H1047R^*-expressing mammary gland of 6 and 12 week old mice but increased pAKT and pS6 were clearly evident in the mutant ducts of adult mice. It is possible that, at early stages of development phosphatases, such as PTEN, or negative feed-back mechanisms may counteract increased signaling induced by mutant *Pik3ca* but that, over time, accumulation of other mutations eventually leads to a reduction in these regulatory mechanisms with subsequent expression of constitutive PI3K/AKT activity and, ultimately, tumorigenesis. Consistent with this hypothesis, we have recently shown that *Pik3ca* ^H1047R^ expression in the mouse ovary does not, by itself, induce cancer but can synergise with *Pten* loss to rapidly induced tumorigenesis [28].

The time required for tumor development observed in our model is significantly longer than that reported for the transgenic *Pik3ca^H1047R^* models [22], [23], [24]. The reasons for this are not clear but it is possible that the exogenous promoters used in the transgenic models may result in higher levels of expression of the mutant protein resulting in the endogenous regulatory mechanisms being overcome.

In contrast to our *in vivo* model where only ductal hyperplasia is observed at 6 weeks, the *in vitro* induction of the *Pik3ca^H1047R^* mutation in immortalized mammary epithelial cells from 6 week old mice resulted in a strong tumorigenic phenotype when injected into the mammary fat pad of immuno-deficient recipient mice. It is noteworthy that tumor formation was not observed in primary cells from either *Pik3ca^H1047R^* mutant or control mammary epithelial cells but only in *Pik3ca^H1047R^* mutant cells that had been immortalized by expression of dominant negative p53 and E1A. A cooperative interaction between *Pik3ca^H1047R^* and p53 loss-of-function has previously been reported in an *in vivo* mouse model of mammary cancer [22]. This raises the possibility that the tumorigenic activity we observed following expression of the *Pik3ca* ^H1047R^ in immortalized cells may result from a cooperation between *Pik3ca^H1047R^* and the dominant-negative p53 used for immortalization and provides further support for our contention that *Pik3ca^H1047R^* alone is insufficient to cause cancer and that other secondary mutational events are required for tumorigenesis.

The tumors formed in response to *Pik3ca^H1047R^* expression were heterogenous in histology forming mainly benign fibroadenomas, sarcomas and carcinosarcomas,. This range in tumor types is similar to the histologies reported in the transgenic *Pik3ca^H1047R^* models [22], [23], [24] and is consistent with the heterogeneity observed in human breast cancer. Since the MMTV-Cre targets a multiple cell types within the mammary gland including both ductal and luminal progenitors [29], [30] the observed tumor heterogeneity possibly reflects different cells of origin. Fibroadenomas and carcinosarcomas expressed both K5 and K8, markers of ductal and luminal cells respectively, suggesting these tumor types may derive from primitive multipotential progenitor cells. The high proportion of ERα-positive tumors could indicate that *Pik3ca^H1047R^* tends to drive transformation down the luminal pathway, a result consistent with our FACS data which showed an expansion of the luminal progenitor population induced by *Pik3ca^H1047R^* in the developing mammary gland. On the other hand, the adenosquamous carcinomas stained positively for K5 but did not express K8 or ERα suggesting they may be derived from a differentiated myoepithelial cell. However, since the expression of differentiation markers in tumor cells is not necessarily indicative of the originating cancer-initiating cells [50], no firm conclusions can be drawn as to the cells of origin in this model.

We have described a novel mouse model that accurately reproduces the scenario of a somatic mutation in a single *PIK3CA* allele as occurs in human breast tumors. Our results confirm that *Pik3ca^H1047R^* mutation, when expressed at endogenous and physiologically relevant levels, predisposes to mammary tumorigenicity. Furthermore, we have demonstrated that this mouse can be used to study the biological consequences of *PIK3CA* mutations both *in vivo* and *in vitro* and, as such, this model will be a powerful tool for investigating the role of *PIK3CA* mutations in tumourigenesis. In addition, this mouse will be a valuable model for the preclinical evaluation of therapies targeting the PI3K pathway.

## Supporting Information

Figure S1 **Immunohistochemical staining of mammary glands.** Sections of formalin-fixed paraffin-embedded mammary tissue from control (MMTV-Cre) and mutant (*Pik3ca* ^H1047R^:MMTV-Cre) mice were stained with anti-smooth muscle actin, anti-p63, anti-K8/18, or anti-β-catenin, antibodies and counter stained with hematoxylin. (A) Smooth muscle actin (SMA) expression of the periductal stroma of 12 week old mice. Arrows indicate periductal SMA staining. (B) p63 expression in the ducts and terminal end buds of 6 week old mice. Arrows indicate examples of p63 staining of non-basal cells. (C) K8/18 expression in the ducts and terminal end buds of 6 week old mice. (D) β-catenin expression in the ducts and terminal end buds of 6 week old mice. All Scale bars are 100 µm. Insets (top left) show lower magnification image of surrounding regions.
(TIF)
