# Supplementary figures and images for: Physiological Levels of Pik3ca H1047R Mutation in the Mouse Mammary Gland Results in Ductal Hyperplasia and Formation of ERα-Positive Tumors
Source: PLoS One. 2012 May 30;7(5):e36924. doi: 10.1371/journal.pone.0036924 (PMC3364244; doi:10.1371/journal.pone.0036924)

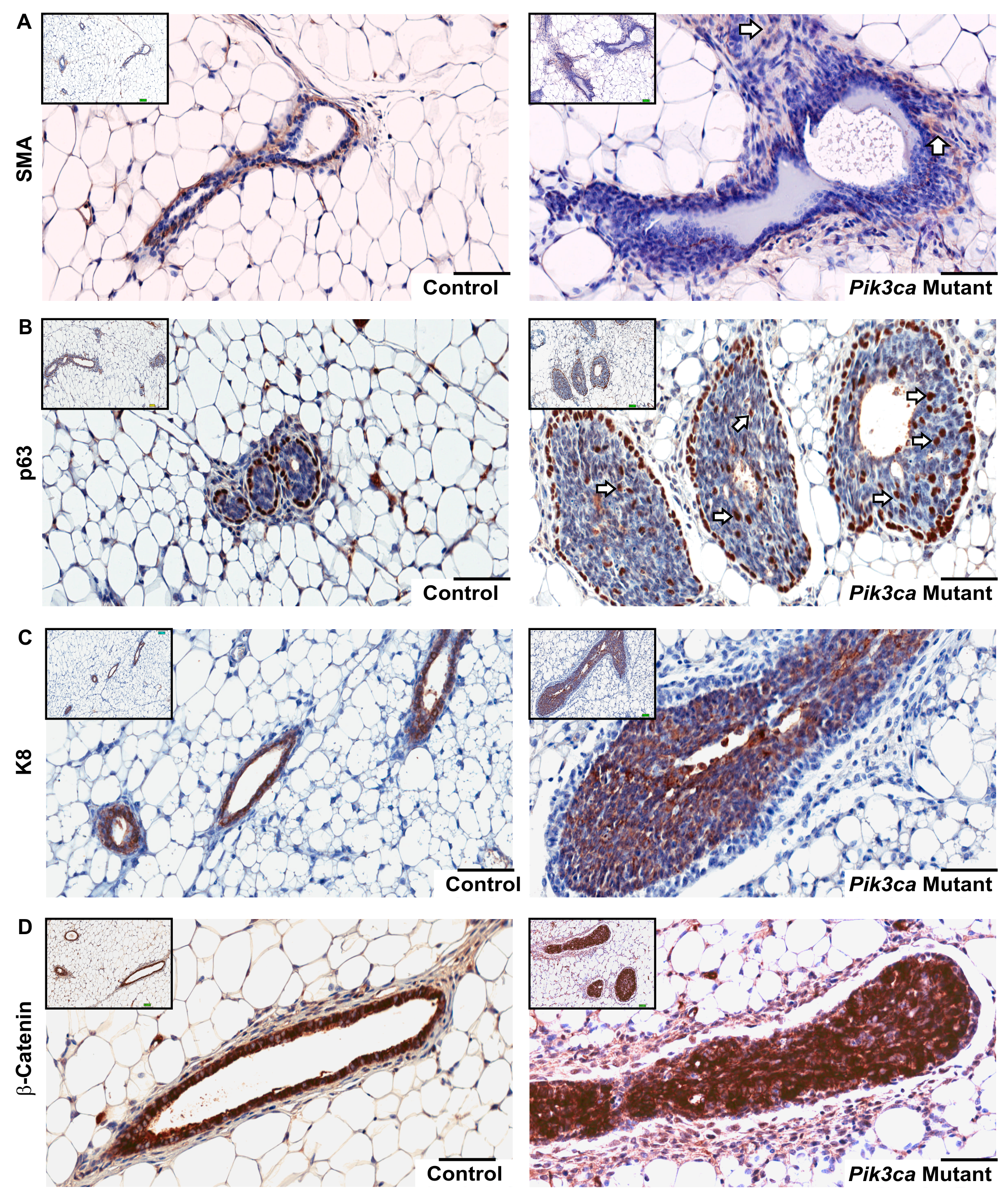

Supplement: Figure S1 — Immunohistochemical staining of mammary glands. Sections of formalin-fixed paraffin-embedded mammary tissue from control (MMTV-Cre) and mutant (Pik3ca H1047R:MMTV-Cre) mice were stained with anti-smooth muscle actin, anti-p63, anti-K8/18, or anti-β-catenin, antibodies and counter stained with hematoxylin. (A) Smooth muscle actin (SMA) expression of the periductal stroma of 12 week old mice. Arrows indicate periductal SMA staining. (B) p63 expression in the ducts and terminal end buds of 6 week old mice. Arrows indicate examples of p63 staining of non-basal cells. (C) K8/18 expression in the ducts and terminal end buds of 6 week old mice. (D) β-catenin expression in the ducts and terminal end buds of 6 week old mice. All Scale bars are 100 µm. Insets (top left) show lower magnification image of surrounding regions. (TIF) [file pone.0036924.s001.tif]
